# Informing Behaviour Change: What Sedentary Behaviours Do Families Perform at Home and How Can They Be Targeted?

**DOI:** 10.3390/ijerph16224565

**Published:** 2019-11-18

**Authors:** Lauren Arundell, Kate Parker, Jo Salmon, Jenny Veitch, Anna Timperio

**Affiliations:** Institute for Physical Activity and Nutrition (IPAN), School of Exercise and Nutrition Sciences, Deakin University, Geelong, VIC 3220, Australia; k.parker@deakin.edu.au (K.P.); jo.salmon@deakin.edu.au (J.S.); jenny.veitch@deakin.edu.au (J.V.); anna.timperio@deakin.edu.au (A.T.)

**Keywords:** sedentary behavior, child, family, feasibility, intervention, prevalence, home environment, adolescent

## Abstract

Home-based interventions targeting children’s sedentary behaviours have had limited and inconsistent effectiveness, possibly due to a mismatch between the behaviours targeted, the behaviours actually performed, and health-risk messages parents need to initiate change. Between October 2017–February 2018, 540 parents completed an online survey indicating their own and their child’s participation in 15 home-based sedentary behaviours (child mean age 11.1 ± 2.61 years, 52% male; parent mean age 40.7 ± 6.14, 93% female). Parents also indicated which home-based sedentary behaviours they and their child could reduce, and what health-risk messages would make them change their child’s behaviours. The most prevalent sedentary behaviours among children (particularly older children) and parents were screen-based leisure-time activities, specifically TV/video/DVD use (67.5 and 62.5 min/day, respectively) and using a tablet/smart phone for leisure (53.6 and 80.8 min/day, respectively). Importantly, these were also perceived as the most feasible behaviours parents and children could reduce. Parents reported that the following messages would help them reduce their child’s sedentary behaviour: sitting may *increase the risk of poor mental health* (85.2% of parents) and *adversely impact future health as an adult* (85.1%). These findings highlight feasible behavioural targets and intervention content for programs aiming to reduce sedentary behaviours in the home environment. Further research is needed to test these strategies.

## 1. Introduction

Growing evidence links sedentary behaviours, defined as any sitting, reclining or lying behaviours requiring an energy expenditure of ≤1.5 metabolic equivalents (METs) [1], with a multitude of negative health outcomes such as an increased risk of obesity, cardio-metabolic risk factors, anti-social behavior, and lower academic achievement [2]. However, sedentary behaviours are ubiquitous and pervasive, making them difficult to change. The home environment has been identified internationally as a key setting and source of influence on children’s overall sedentary behaviour levels [3,4,5]. To date, home-based interventions aiming to reduce children’s recreational electronic media use have primarily focused on managing TV viewing, with limited and inconsistent results [6,7,8]. This is likely due to the rapid change in the technology environment and the proliferation of “new” technologies [9] such as digital tablets, smart phones, game consoles, and readily available internet. The lack of intervention effects may, therefore, be due to a mismatch between sedentary behaviours targeted in the interventions and the range of sedentary behaviours children perform at home. For example, strategies previously trialled to target home-based sedentary time among children have been predominantly from the United States of America and New Zealand and include TV monitoring or locking devices, active video games, and feed-back systems where children can view TV only while peddling a bike have shown mixed and limited effectiveness at reducing sedentary behaviour [6]. This is potentially because the strategies were not targeting the wide range of sedentary behaviours that children currently perform.

The types of sedentary behaviours children engage in appear to be changing, in line with the availability of new technologies. International data from 30 European countries exploring trends in screen time, TV viewing, and computer use showed an increase in overall screen time from 2002 to 2010 [10]. However, when screen behaviours were examined separately, a decline in TV viewing and an increase in computer use was observed over this time, particularly amongst boys [10]. This trend was mirrored among adolescents in the US from 2002 to 2015 [11]. Over this 13-year period, there was a significant decline in daily TV viewing (from 4.1 to 3.3 h per day) accompanied by an increase in computer use (from 3.2 to 4.0 h/day) [11]. Changes amongst Australian populations are similar. For example, between 2009 and 2012, there was a small reduction in the proportion of children watching TV, DVD or videos, and an increase in the proportion engaging in computer, internet, and games use [12]. There is, however, a current lack of understanding of how children use other technologies such as digital tablets and smartphones, which have increased in prevalence [12] and, therefore, may have become important intervention targets.

In addition to screen-based sedentary behaviours, there is evidence that children also engage in large amounts of non-screen based sedentary behaviours in the home, such as sitting and talking with friends [13,14], which may also serve as behaviours to change through intervention. Australian [15] and Canadian [16] sedentary behaviour guidelines state children (5 to <18 years) should limit their recreational screen use to less than two-hours per day and break up long periods of sitting as often as possible. Therefore, it is important to understand whether the modifiable sedentary behaviours children perform are during their recreational time.

Participatory research methods and design [17,18] may be important for the development of intervention strategies targeting sedentary behaviours. Participatory research utilises end-users to guide and inform the development of interventions that be offered to them [17,18]. They incorporate a ‘bottom-up’ approach, whereby local knowledge and perspectives shape the structure and content of the research or intervention program [18]. Simply put, interventions are designed by end-users for use by end-users. To inform the development of future behaviour change strategies targeting a wide variety of home-based sedentary behaviours, it is important to gain an understanding of not only the behaviours being performed, but also the behaviours that parents and children consider feasible to reduce. Such insight would provide a basis for key intervention targets. Further, understanding what informational prompts parents need to help them initiate change would underpin intervention messages and materials. This study aims to: (1) describe the time that Australian children and parents spend engaged in a variety of sedentary behaviours in the home, including sex and age differences in children’s behaviours, (2) identify the sedentary behaviours parents perceive they and their child could reduce, and (3) examine the likelihood of parents initiating behaviour change based on different health-related consequences of sitting.

## 2. Materials and Methods

### 2.1. Sample

Parents of children aged 8–16 years living in Australia were recruited to the Sitting In the Home (SIT) Study from October 2017 to February 2018 through social media (Facebook and Instagram) and snowball techniques. After completing a screening questionnaire (to ensure they reside in Australia and have a child 8–16 years, participants were directed to the online survey. In total, 1925 parents clicked on the study link, 1587 progressed past the screening questions to commence the study, and 553 parents (29%) completed the survey. The study was conducted in accordance with the Declaration of Helsinki. Ethical approval was obtained from the Deakin University Human Ethics Advisory Group-Health (HEAG-H 123_2017), and informed consent from participants was obtained at the start of the survey. Upon completion of the survey, participants received an AU$10 e-gift card as compensation for their time.

### 2.2. Measures

Participants provided information about their own and their child’s age and sex. If the parent had multiple eligible children, they were asked to consider the child with the next birthday. Children were dichotomized into two groups: younger children 8–11 years and older children 12–16 years. This approximately reflects primary (elementary) and secondary school-aged groups. Parents also reported their highest level of schooling, marital status, and if English is the main language spoken at home.

Parents proxy-reported their child’s participation (yes/no) in 15 sedentary behaviours at home while sitting during a typical school week, excluding school holidays. These included: watching TV/videos/DVDs, using a tablet/smart phone for leisure, using the computer/laptop for leisure, talking to others (in person or on the phone), playing or listening to music, completing paperwork/writing/studying/homework, using the computer/laptop for homework/work, reading for leisure, using game consoles, reading for homework/work, pretend play (e.g., dolls and trains etc.), doing artwork or crafts, completing chores, using a tablet/smart phone for homework/work, and preparing and cooking food. Parents then reported their child’s total minutes spent engaging in these behaviours on an average week day and average weekend day. Behaviours that were not performed were coded as 0 min. Parents then completed the same questions to self-report their sedentary behaviours at home.

Average day duration for each behaviour, for screen-based sedentary behaviours, non-screen-based sedentary behaviours, and total sedentary behaviours were calculated as [(weekday time in Xbehaviour × 5) + (weekend day time in Xbehaviour × 2)]/7. Inferential statistics were determined as mean ± standard deviation. The average time spent in these behaviours on weekdays and weekend days was determined, and differences assessed via paired samples *t*-tests. The percentage of children meeting the current screen-based sedentary behaviour guidelines was calculated by summing the average day duration of time spent in screen-based behaviours during leisure-time (watching TV/videos/DVDs, using a tablet/smart phone for leisure, using the computer/laptop for leisure and using game consoles). Differences between younger and older children were examined using chi-square tests.

To identify feasible intervention targets, parents were provided with the list of 15 sedentary behaviours (described above) and asked which they thought their child could reduce if they were trying to decrease the amount of time they spend sitting at home (response options for each behavior: yes definitely could reduce, maybe, no, doesn’t apply to my child). Amongst those who participated in each sedentary behaviour, the percentage of participants who could change their time spent in that behaviour was determined. Participants responding “*doesn’t apply to me*” were not included in the analysis. Parents then self-reported which behaviours they could reduce if trying to reduce their own sitting time at home.

To better understand what would motivate parents to initiate a reduction in their child’s home-based sedentary behaviours, parents were asked to respond how likely (4-point Likert scale: very likely to very unlikely) they would be to try to reduce their child’s sitting at home if they were shown eight statements, “How much your child sits now can increase their risk for (1) diabetes and cardiovascular disease, (2) overweight and obesity, (3) poor muscle and bone health, (4) poor mental health, and adversely impact their (5) academic outcomes, (6) social skills, (7) level of resilience, and (8) future health as an adult”. Responses were collapsed into “likely” (combined *very likely* and *likely*) and “unlikely” (combined *unlikely* and *very unlikely*) and the percentage of participants in each group reported.

### 2.3. Data Management and Analysis

Data were analysed using STATA SE version 15 (StataCorp LP, College Station, TX, USA). Duration of sedentary behaviours at home, the feasibility of reducing these behaviours, and the likelihood of taking action based on different consequences of sitting were examined using descriptive statistics. Sex and age group differences in child home-based sedentary behaviours were determined using independent sample *t*-tests with significance set at *p* < 0.05. Sex differences in behaviours performed by parents could not be determined as the majority of respondents were female.

## 3. Results

The final sample consisted of 540 parents who provided data for the variables of interest. On average, children were aged 11.1 (±2.6) years (range: 8–16 years), and 52% were boys. Parents’ mean age was 40.7 (±6.1) years, and 93% were women. The majority of parents had a university or tertiary qualification (63.6%) and spoke English at home (99%).

### 3.1. Prevalence of Parent and Child Home-Based Sitting Behaviours

Table 1 shows the average minutes per day that the children and parents typically spent engaged in the 15 sedentary behaviours at home with the wide standard deviations highlighting the variance in participation levels amongst participants. Among children, the three most prevalent behaviours were screen-based leisure-time activities, including watching TV/videos/DVDs, using a tablet/smart phone, and using the computer/laptop. The top three non-screen-based sedentary behaviours at home included talking to others (in person or on the phone), playing or listening to music, and completing paperwork/writing/studying/homework. Among parents, the three most prevalent behaviours were using a tablet/smartphone for leisure, watching TV/videos/DVDs, and completing chores. On average, children and parents engaged in sedentary behaviours for 7 h and 7 min and 5 h and 5 min per day, respectively. Screen-based sedentary behaviours averaged 3 h and 38 min for children and 3 h and 40 min per day for parents, and non-screen-based sedentary behaviours 3 h and 18 min and 3 h and 38 min per day, respectively. In total, 65% of children exceeded the screen-based sedentary behaviour guidelines with significantly more older children (78%) exceeding the recommendations than younger children (56%; *p* < 0.01).

**Figure 1:**
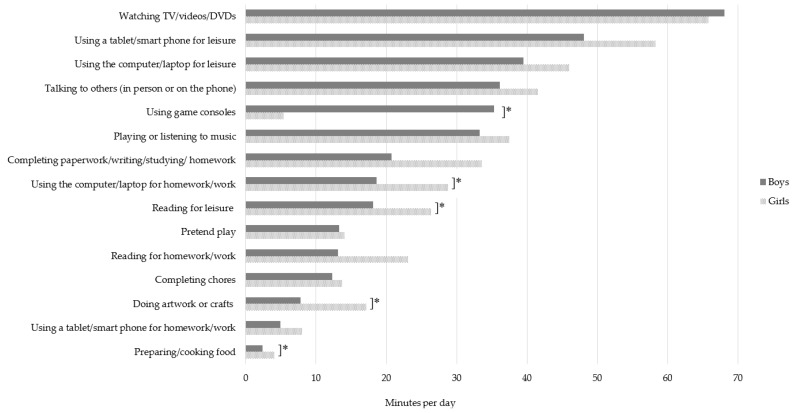
Total duration (minutes/day) of home-based sitting behaviours by sex. * Significant differences between boys and girls determined by independent samples *t*-tests, *p* < 0.05.

Sex differences in daily time children spent engaged in five sedentary behaviours were observed (Figure 1). Boys participated in greater amounts of game console use than girls (35.3 vs. 5.5 min/day respectively), and girls participated in greater amounts of computer/laptop use for homework than boys (31 vs. 18.6 min/day), reading for leisure (29.2 vs. 18.1 min/day), artwork and craft (18.9 vs. 7.8 min/day), and preparing and cooking food (4.1 vs. 2.4 min/day), respectively. Girls spent significantly more time than boys in non-screen based sedentary behaviours (165.88 vs. 121.5 min/day respectively, *p* = 0.01). There were no significant differences between boys and girls for total sitting behaviours (288.7 vs. 341.7 min/day, respectively; *p* = 0.053), or total screen-based sitting behaviours (174.4 vs. 183.3 min/day, respectively; *p* = 0.56).

**Figure 2:**
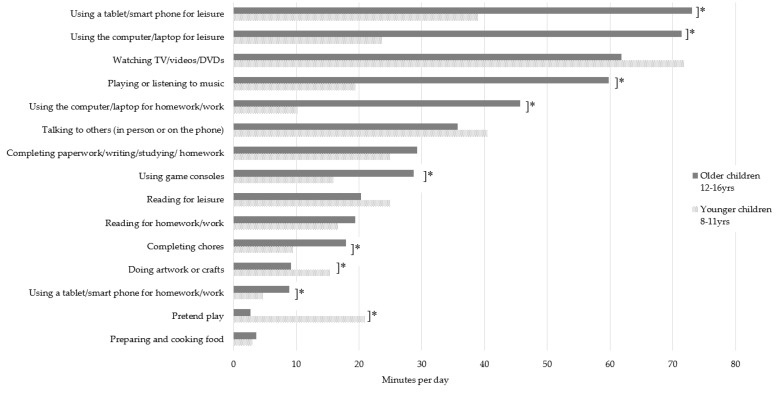
Total duration (minutes/day) of children’s home-based sitting behaviours by sex group. * Significant differences between older and younger children determined by independent samples *t*-tests, *p* < 0.05.

Age group differences in children’s daily sedentary behaviours are shown in Figure 2. There were age group differences in nine of the sedentary behaviours. Older children spent more time than younger children using a tablet/smart phone for leisure (73.2 vs. 40 min/day), using the computer/laptop for leisure (71.8 vs. 23.6 min/day), using games consoles (29 vs. 16 min/day), using the computer/laptop for homework (45.7 vs. 10.3 min/day), using the tablet/smart phone for homework (8.9 vs. 4.8 min/day), playing or listening to music (59.8 vs. 19.4 min/day), and completing chores (18 vs. 9.6 min/day), respectively. Younger children spent more time than older children doing pretend play (20.9 vs. 2.7 min/day) and doing artwork and crafts (15.5 vs. 9.1 min/day), respectively. Compared to younger children, older children spent more time in total sedentary behaviours (391.2 vs. 262.6 min/day respectively, *p* < 0.001) and total screen-based sedentary behaviours (244.1 vs. 135.5 min/day respectively, *p* < 0.001). There were no significant differences between older and younger children’s non-screen based sedentary behaviours. Amongst children and parents, there were significant differences in time spent in sedentary behaviours between weekdays and weekend days for all but three sedentary behaviours (see Table S1). Compared to weekdays, on weekend days parents and children spent more time engaged in total sitting (children: 580.1 vs. 350 min/day; parents: 598.2 vs. 459.1 min/day, respectively), total screen-based sitting (children: 318.5 vs. 194.9 min/day; parents: 322.0 vs. 264.4 min/day, respectively) and total non-screen-based sitting behaviours (children: 249.7 vs. 167.8 min/day; parents: 263.1 vs. 197.0 min/day, respectively).

### 3.2. Sedentary Behaviours Children and Parents Could Change

The sedentary behaviours that parents felt they or their child could “definitely” or “maybe” reduce are shown in Table 2. More than 50% of parents thought they and their child could “definitely” reduce the time they spent siting using a tablet/smart phone for leisure, watching TV/videos/DVDs, and using the computer/laptop for leisure. In addition, 53% of parents thought their child could reduce the time they spend sitting using games consoles.

### 3.3. Impacts of Sedentary Behaviour on Child Health

Table 3 shows the percentage of parents who reported that they would be likely to try to reduce their child’s home-based sedentary behaviours if they were told of the impact of sitting on their child’s health. Across the eight statements, the proportion who reported that they would try to initiate change ranged from 78 to 85%. The statements with the highest reported likelihood of action were that sitting too much could increase their child’s risk of poor mental health (85%), adversely impact future health as an adult (85%), increase risk for poor muscle and bone health (83%) and increase risk factors for diabetes and cardiovascular disease (81%). All statements were considered important for motivating change by at least 78% of parents.

## 4. Discussion

This study explored the sedentary behaviours performed by children and parents within the home setting and provides important information regarding key behavioural targets for interventions and the impetus parents may need to prompt them to initiate behaviour change. This study provides novel and timely evidence that can be used to inform the development of effective intervention strategies targeting sedentary behaviours in the home.

Consistent with previous research examining home-based sedentary behaviours [13], both screen and non-screen sedentary pastimes were prevalent, particularly on weekend days. However, the large majority of time was spent engaged in leisure-time screen-based activities. Overall, only one-third of participants in the current study met the screen-based sedentary behaviour recommendation of less than two hours per day [15]. However, when examined according to age group, a greater percentage of children (44%) and adolescents (22%) in the current study achieved these recommendations compared to Australian national data (children 35% and adolescents 20%). On average, children and parents engaged in more than 60 min of TV/video/DVD viewing per day, with high levels of tablet/smartphone and computer/laptop use for leisure, was also reported. These findings add Australian data to the literature that highlights excessive screen time is evident internationally. The average daily duration of combined TV viewing and computer use reported in 30 children/adolescent studies published between 2012–2016 was 2.9 h/day, and from 39 adult studies was 2.5 h/day [19]. The current findings also highlight that in addition to these traditional technologies, children and parents spend large amounts of time using newer technologies (e.g., tablets and smartphones). Therefore, there is considerable scope to reduce sedentary behaviours in the home. Interventions may also seek to target weekend days as children and parents are likely to be at home for longer than during weekdays, and more of their time is spent in both screen- and non-screen-based sedentary behaviours. There may also be different influences on week day and weekend day behaviours. For example, the presence of rules is consistently inversely associated with screen time (TV viewing and computer use) [20]. However, there is also evidence to suggest that rules restricting sedentary activities are more influential on weekday activity compared to weekend activity [21]. Parents may, therefore, need additional encouragement and support to enforce rules on weekends. Given the similar patterns in children’s and parents’ sitting behaviours, interventions should also include strategies that target reducing both child and parent sitting [7].

A novel contribution of this study is the combination of data on sedentary behaviour participation with data on the perceived feasibility of reducing time spent in these behaviours. The high percentage of parents who indicated they would try to reduce their child’s sedentary behaviours upon learning of the associated health risks, suggests that parents are unaware of the risk of elevated sitting time. It appears that parents are most concerned about the impact on their child’s mental health and future health as an adult, and therefore messages about these health risks should be included in the development and/or promotion of interventions. The findings also showed that the most common behaviours that parents believed they and their child could reduce were leisure-time screen-based activities (tablets/smartphones, using game consoles TV/videos/DVDs, and computer/laptops). Importantly, the top behaviours considered feasible to change were the same among both children and parents, which suggests that family-based intervention strategies to reduce these behaviours may be well received and potentially effective.

Given that parents play a pivotal role in children’s sedentary behaviour participation through behavioural role modeling and co-participation, targeting reductions in their sedentary behaviour is also an important intervention target [22,23,24]. A systematic review of family-based interventions to reduce children’s sedentary time found that despite inconsistent results regarding reductions to sedentary time, interventions with greater parental involvement appeared to be more effective [6]. While the focus of these interventions was primarily TV viewing, parental involvement and role modelling may be equally important for other screen- and non-screen based sedentary behaviours [25]. Focusing on parental behaviours may, therefore, result in improvements to both children’s and parents’ sedentary time. Overall, the combined prevalence and feasibility data in the current study highlights the importance of participatory research methods in the design of interventions targeting home-based sedentary behaviours [17,18] as these findings may directly inform intervention development and content.

Findings from the current study also showed that in addition to leisure-time sedentary behaviours (and chores amongst parents), children, particularly the older children, and parents performed various sedentary behaviours for homework or work pursuits. Targeting reductions in these behaviours may prove difficult due to the increasing requirements from schools or workplaces and the positive associations between completing homework and reading and higher academic achievement [26]. Alternatively, intervention strategies could instead encourage breaking up long periods of sitting while performing these activities. This may result in improvements to health for children [27] and parents [28] as well as assist them in achieving recommendations to reduce and break up long periods of sitting [15,16].

The sex differences in sedentary behaviours, particularly game console use, are similar to what has been previously reported [14]. Among the current sample, boys spent an additional 30 min per day using game consoles compared to girls. This is consistent with the findings from a large sample (n = 1513) of 9–10-year-old children from the United Kingdom, which found that boys and girls played videogames for the equivalent of 36 and 11 min/day, respectively [14]. Conversely, girls in the current study spent an additional 44 min/day in non-screen based sedentary behaviours and 12 more mins/day than boys using the computer/laptop for homework and reading for leisure. Importantly, participation in these behaviours is associated with different health outcomes. For example, elevated computer game use is associated with anti-social behaviour whereas time spent reading is beneficial for academic outcomes [26]. Interestingly, there was no difference between boys’ and girls’ total sedentary behaviour or screen-based sedentary behaviour time. This highlights the need to examine the individual behaviours as differences can be hidden when combining them to create a total daily duration. Interventions targeting children’s sedentary behaviours may require sex- and behaviour-specific intervention strategies.

The differences in sedentary behaviours between younger and older children are consistent with previous literature showing that age is associated with greater sedentary behaviour and sedentary time [13,29]. The current study extends this knowledge by highlighting the types of behaviours performed. Older children engaged in greater amounts of sedentary behaviours which are primarily screen-based, and are performed for leisure and homework. Interventions may, therefore, require unique strategies for primary- and secondary-school aged children and parents to target their differing behaviours. Further, using anticipatory guidance methods [30], strategies for parents of younger children may focus on preparing and equipping them with the skills and knowledge needed to manage the imminent changes in their child’s sedentary behaviour at home [31].

The strengths and limitations of the current study should be considered when interpreting the results. Limitations include the use of subjective measures, including proxy-report of children’s sedentary behaviours. Season, participant ethnicity, and the existence of any impairment that limits movement activity were not examined but would be important to include in future research examining correlates of home based sedentary behaviour. Future research could also examine if participation in home based sedentary behaviours, the behaviours perceived as feasible to change, and the required health messages to motivate behaviour change, vary according to socio demographic factors. The survey did not capture behaviour multitasking, where more than one sedentary behaviour may be performed simultaneously. Therefore, the combined time spent in sedentary behaviour may overestimate the sedentary time and screen time. There may also be other sedentary behaviours not examined that parents and children participate in, which may be potential intervention targets. The strengths of this study include the large sample of Australian children and parents which provides evidence that the well-educated, mainly English speaking sample had a high prevalence of home-based sedentary behaviours. This is consistent with previous literature showing higher socio-economic status (SES) is associated with more sedentary behaviour [29]. Further, to our knowledge, this is the first study that has examined such a wide variety of home-based sedentary behaviours and differentiated between home/work and leisure-time activities. Valuable and novel information for the development of interventions specifically targeting sedentary behaviours that children and parents perform at home has been identified.

## 5. Conclusions

In conclusion, this study indicates that sedentary behaviours, particularly screen-based leisure-time activities performed in the home environment, are highly prevalent amongst children and parents. Importantly, they are also the behaviours parents perceive that they and their child could reduce. The provision of information pertaining to mental health outcomes and future health implications of excessive sitting time may be an important stimulus for parents to initiate changes in their child’s home-based sedentary behaviours. These findings provide participant-derived information for the development of interventions targeting children’s and parents’ home-based sedentary behaviours.

## Figures and Tables

**Table 1:** Mean daily duration (minutes/day ± standard deviation (SD)) of home-based sitting behaviours by children and parents.

	Children		Parents	
	n	Mean mins/day (±SD)	n	Mean mins/day (±SD)
Watching TV/videos/DVDs	430	67.5 (±59.3)	397	62.5 (±64.9)
Using a tablet/smart phone for leisure	424	53.6 (±70.3)	425	80.8 (±104.2)
Using the computer/laptop for leisure	434	42.7 (±72.0)	432	38.3 (±63.7)
Talking to others (in person or on the phone)	397	39.3 (±72.4)	410	47.4 (±79.1)
Playing or listening to music	400	35.9 (±69.9)	418	27.7 (±88.5)
Completing paperwork/writing/studying/homework	364	26.7 (±65.2)	403	13.4 (±42.4)
Using the computer/laptop for homework/work	456	24.5 (±57.7)	426	47.2 (±91.7)
Reading for leisure	386	23.2 (±32.8)	405	25.9 (±49.2)
Using game consoles	404	21.1 (±50.0)	426	1.5 (±12.7)
Reading for homework/work	355	17.7 (±49.8)	414	9.1 (±35.1)
Pretend play	379	13.6 (±26.7)	415	3.1 (±29.6)
Doing artwork or crafts	369	13.0 (±24.3)	414	7.4 (±28.3)
Completing chores	395	13.0 (±35.0)	416	52.8 (±99.6)
Using a tablet/smart phone for homework/work	404	3.7 (±19.7)	414	11.9 (±30.1)
Preparing and cooking food	387	3.2 (±7.8)	427	25.5 (±65.2)
Total sedentary behaviours	192	427.3 (±264.3)	250	485.2 (±530.3)
Total screen-based sedentary behaviours	290	218.0 (±165.1)	324	250.7 (±233.2)
Total non-screen-based sedentary behaviours	217	197.7 (±164.1)	290	218.2 (±337.2)

**Table 2:** The percentage of parents who reported they or their child could decrease the time they spend in various sedentary behaviours.

	Children		Parents	
If Trying to Reduce Home-Based Sitting, Could You/Your Child Reduce Time Spent…	Yes, Definitely Could Reduce (%)	Maybe Could Reduce (%)	Yes, Definitely Could Reduce (%)	Maybe Could Reduce (%)
Using a tablet/smart phone for leisure	58.9	31.8	64.5	26.3
Using game consoles	52.6	28.2	38.5	23.9
Watching TV/videos/DVDs	50.2	37.7	52.2	30.5
Using the computer/laptop for leisure	50.5	35.3	53.2	28.6
Using a tablet/smart phone for homework/work	20.1	23.2	35.4	25.1
Using the computer/laptop for homework/work	15.9	24.1	32.7	21.9
Playing or listening to music	7.4	29.6	22.2	27.1
Talking to others (in person or on the phone)	6.9	24.1	27.9	31.9
Pretend play	6.9	19.9	26.1	17.9
Completing paperwork/writing/studying/homework	5.4	20.7	19.7	24.9
Doing artwork or crafts	4.5	20.7	23.9	23.0
Completing chores	6.6	14.5	23.0	20.1
Reading for leisure	3.8	16.7	17.1	24.0
Preparing and cooking food	5.5	15.0	19.8	13.6
Reading for homework/work	3.5	14.3	18.9	23.3

**Table 3:** Percentage of parents who would try to reduce their child’s sitting based on health risk statements.

Statement: Would You Try to Reduce Your Child’s Sitting at Home If Told Sitting Too Much Could…	% Parents *Likely* to Try to Reduce Child’s Sitting
Increase their risk for poor mental health	85.2
Adversely impact future health as an adult	85.1
Increase their risk for poor muscle and bone health	82.7
Increase their risk factors for diabetes and cardiovascular disease risk	81.4
Increase their risk for overweight and obesity	79.1
Adversely impact academic outcomes	79.1
Adversely impact their social skills	78.5
Adversely impact their level of resilience	77.6

Note: ‘*Likely*’ is the sum of survey responses ‘*very likely*’ and ‘*likely*’.

## Supplementary Materials

The following are available online at https://www.mdpi.com/1660-4601/16/22/4565/s1, Table S1: Average (minutes/day) weekday and weekend day sitting behaviours amongst children and parents.
