# Supplementary material for: Informing Behaviour Change: What Sedentary Behaviours Do Families Perform at Home and How Can They Be Targeted?
Source: Int J Environ Res Public Health. 2019 Nov 18;16(22):4565. doi: 10.3390/ijerph16224565 (PMC6888231; doi:10.3390/ijerph16224565)
Supplement: Supplementary file 1 [file ijerph-16-04565-s001.pdf]

Table S1: Average (minutes/day) weekday and weekend day sitting behaviours amongst children and parents

	Children			Parents		
	Weekday Mean mins/day (±SD)	Weekend day Mean mins/day (±SD)	p-value	Weekday Mean mins/day (±SD)	Weekend day Mean mins/day (±SD)	p-value
Watching TV/videos/DVDs	53.8 (±52.6)	**102.1 (±96.9)**	**<0.001**	82.9 (85.0)	**109.9 (99.2)**	**<0.001**
Using a tablet/smart phone for leisure	43.3 (±61.4)	**76.3 (±100.8)**	**<0.001**	75.4 (110.0)	**97.0 (125.9)**	**<0.001**
Using the computer/laptop for leisure	35.5 (±68.3)	**67.8 (±110.6)**	**<0.001**	33.3 (59.8)	**44.1 (89.9)**	**<0.001**
Talking to others (in person or on the phone)	35.3 (±71.3)	**54.3 (±98.5)**	**<0.001**	45.6 (93.7)	**57.7 (95.5)**	**<0.001**
Playing or listening to music	32.0 (±72.9)	**52.2 (±105.9)**	**<0.001**	25.5 (87.0)	**35.2 (104.6)**	**0.002**
Completing paperwork/writing/studying/ homework	**29.5 (±65.7)**	19.3 (±61.2)	**<0.001**	14.4 (41.0)	15.7 (57.5)	0.409
Using the computer/laptop for homework/work	25.3 (±58.5)	23.8 (±68.7)	0.634	**54.7 (107.9)**	40.4 (82.7)	**0.013**
Reading for leisure	21.3 (±29.9)	**30.7 (±45.1)**	**<0.001**	25.2 (47.8)	**35.1 (65.1)**	**<0.001**
Using game consoles	16.8 (±43.1)	**40.4 (±83.9)**	**<0.001**	1.1 (7.9)	3.2 (28.3)	**0.043**
Reading for homework/work	18.6 (±41.9)	17.3 (±65.3)	0.551	10.3 (31.7)	10.1 (55.7)	0.574
Pretend play	11.0 (±21.4	**23.3 (±44.3)**	**<0.001**	**5.6 (62.8)**	3.7 (30.7)	**0.017**
Doing artwork or crafts	10.7 (±21.3)	**22.0 (±37.6)**	**<0.001**	7.8 (29.4)	**12.1 (46.44)**	**0.002**
Completing chores	11.7 (±31.9)	**17.6 (±43.9)**	**<0.001**	50.0 (100.0)	**67.9 (126.3)**	**<0.001**
Using a tablet/smart phone for homework/work	9.2 (±27.0)	5.8 (±22.7)	0.173	16.5 (62.8)	12.1 (40.0)	0.893
Preparing and cooking food	3.8 (±12.4)	**5.1 (±12.1)**	**<0.001**	27.0 (71.7)	**30.0 (70.1)**	**<0.001**
Total sitting behaviours	350.2 (±246.2)	**580.1 (380)**	**<0.001**	459.1 (461.3)	**598.2 (711.1)**	**<0.001**
Total screen-based sitting behaviours	173.9 (±142.9)	**318.5 (±241.6)**	**<0.001**	264.6 (250.4)	**322.0 (300.9)**	**<0.001**
Total non-screen-based sitting behaviours	167.8 (±159.1)	**249.7 (±221.8)**	**<0.001**	197.0 (287.3)	**263.1 (447.6)**	**<0.001**

Differences between weekdays and weekend days examined by paired samples t-test, significant differences in bold.
